# A systematic review of tumor necrosis factor-α blockers, anti-interleukins, and small molecule inhibitors for dissecting cellulitis of the scalp treatment

**DOI:** 10.1186/s13023-025-03720-5

**Published:** 2025-05-18

**Authors:** Nazila Heidari, Rad Ghannadzadeh Kermani Pour, Melina Farshbafnadi, Amirhossein Heidari, Yekta Ghane

**Affiliations:** 1 School of Medicine, Iran University of Medical Sciences, Tehran, Iran; 2 School of Medicine, Tehran University of Medical Sciences, Tehran, Iran; 3 Faculty of Medicine, Tehran Medical Sciences, Islamic Azad University, Tehran, Iran

**Keywords:** Dissecting cellulitis of the scalp, Hoffman disease, Perifolliculitis capitis abscedens et suffodiens, Cicatricial alopecia, Tumor necrosis factor-α, Interleukin, Janus kinase, Apremilast, Systematic review

## Abstract

**Background:**

Dissecting cellulitis of the scalp (DCS) is a type of neutrophilic scarring alopecia identified by the development of folliculitis with clusters of perifollicular pustules and then progresses to abscesses and intercommunicating sinus formation. In the absence of evidence-based guidelines, the treatment of DCS remains a therapeutic challenge. Our study aimed to assess the safety and efficacy of biologics, including tumor necrosis factor-α (TNF-α) blockers, anti-interleukins (ILs), and small molecule inhibitors, including Janus kinase (JAK) inhibitors and phosphodiesterase inhibitors in treating DCS.

**Methods:**

PubMed/Medline, Scopus, and Ovid Embase databases were systematically searched until February 4th, 2024. Study selection was restricted to case reports, case series, cohort studies, and clinical trials published in English-language. NIH and Murad et al.’s quality assessment tools were utilized for critical appraisal.

**Results:**

A total of 34 articles involving 81 patients met the inclusion criteria. The immunomodulators studied for the treatment of DCS include adalimumab, infliximab, certolizumab pegol, ustekinumab, secukinumab, guselkumab, risankizumab, tildrakizumab, apremilast, upadacitinib, and baricitinib. Our findings implied that TNF-α blockers and IL inhibitors were associated with clinical improvement in most individuals with moderate-to-severe DCS, especially in those who had failed earlier treatments. Moreover, certolizumab pegol could be a safe option for DCS in pregnancy. In addition, the prescription of small molecule inhibitors, including JAK inhibitors and apremilast in DCS patients, demonstrated a significant amelioration in DCS symptoms with a desirable safety profile. Nevertheless, the available data was limited, warranting further investigation. Besides, all aforementioned immunomodulators are still debated for their effectiveness on hair regrowth and reversing the scarring process.

**Conclusions:**

The application of immunomodulators in treating DCS was associated with satisfactory outcomes, although there is still a need to assess the long-term safety and effectiveness of these therapeutic agents in preventing disease progression and new flare-ups.

**Supplementary Information:**

The online version contains supplementary material available at 10.1186/s13023-025-03720-5.

## Highlights

- Dissecting cellulitis of the scalp (DCS) is a rare, refractory skin disease that manifests with a widespread infiltrate of neutrophils and lymphocytes, which turn into papules, pustules, and abscesses, interconnecting sinuses, and fistulas, and eventually, develops scarring alopecia.
- A combination of antibiotics, corticosteroids, retinoids, and surgical interventions have been utilized to alleviate DCS symptoms.
- Similar to hidradenitis suppurativa, DCS pathogenesis is triggered by inflammatory cascades supported by cytokines such as interleukin (IL)-1b, IL-17, IL-23, IL-10, tumor factor necrosis alpha (TNF-α), and the JAK/STAT signaling pathway.
- Immunomodulatory agents, including JAK inhibitors, TNF-α antagonists, IL inhibitors, and phosphodiesterase inhibitors, represent a therapeutic potential for individuals afflicted with DCS due to their notable efficacy and limited adverse events.

**Supplementary Information:**

The online version contains supplementary material available at 10.1186/s13023-025-03720-5.

## Background

Dissecting cellulitis of the scalp (DCS), known as perifolliculitis capitis abscedens et suffodiens or Hoffman’s disease, represents an orphan disease and uncommon form (1 up to 2%) of neutrophilic cicatricial alopecia [1, 2]. DCS primarily afflicts young males of African-American descent during their second to fourth decades of life [3]. The initial pathologic process involves pilar infundibulum blockade arising from hyperkeratosis, retention of obstructed follicular components following rupture, and inducing an extreme inflammatory response at the bulb of the hair follicle [4]. These sequences lead to the formation of pustules that evolve into interconnected tracts, eventually resulting in the development of keloid scar and patchy areas of alopecia [5]. Histopathological examination unveils early lesions identified by dense neutrophilic, lymphocytic, histiocytic, and plasma cell infiltration [6]. DCS exhibits a spectrum of involvement, ranging from isolated scalp area to the entire scalp, with a predominance of the posterior vertex and upper occiput [1, 5].

DCS is recognized as a constituent of the follicular occlusion triad or tetrad (FOT), alongside hidradenitis suppurativa (HS), acne conglobata (AC), and pilonidal disease [5, 6]. DCS and HS can be explained as variant localizations of a similar disease as they share many genetics and environmental factors commonalities [7]. Both genetics and hormonal factors, as well as bacterial propagations, have claimed to possess a crucial role in the pathology of HS and DCS [8]. Additionally, Although the exact pathophysiological pathway of DCS is still unclear, there are several similarities between DCS and HS regarding probable pathogenesis and treatments [9]. HS lesions have exhibited elevated levels of inflammatory cytokines, such as tumor necrosis factor-alpha (TNF-α) and various interleukins (ILs), representing novel potential targets for treatment [10]. It has been postulated that the primary pathogenic event leading to HS is infundibular hyperplasia that proceeds to intrinsic keratinocyte defect. Furthermore, cyst formation and rupture are distinguished by infiltration of neutrophils, macrophages, dendritic cells, and T and B cells. Additionally, the expression of cytokines such as IL-1β, IL-17, and TNF-α is also induced [11]. Moreover, several transcriptomic studies have depicted an upregulation in Janus kinases (JAKs) and subsequent induction of signal transducer and activator of transcription (STAT) activity [12, 13]. TNF-α is a cytokine involved in the pathogenesis of certain inflammatory and autoimmune conditions. Immunomodulator agents act as antagonists by inhibiting the interaction between TNF-α and its type 1 (TNFR1) and 2 (TNFR2) receptors. Additionally, ILs are cytokines first thought to be produced only by leukocytes but have been discovered to be produced by many other body cells [11]. ILs modulate a variety of actions during inflammatory and immune conditions, including growth, differentiation, and activation responses. The cathelicidin gene is responsible for the production of an antimicrobial protein, hCAP18, which is processed into various peptides, including LL-37. Cutaneous inflammation triggers LL-37 production. It has been observed that LL-37 production is amplified in the skin of HS patients. LL-37 is able to attract CD4 T cells and dendritic cells which in turn results in the release of TNF-α, IL-6, and IL-12, which leads to T helper (Th)1/Th17 phenotype, which is not dependent on antigen-presenting cells. HS severity is also directly related to increased cytokine production promoted by LL-37, including IL-17 and TNF-α.

Furthermore, histological findings of both disorders have indicated the accumulation of dense neutrophils, CD3 + T cells (CD4 + and CD8 +), histiocytes, and plasma cells in the early stages of lesion formation, and granulomas, scarring, and fibrosis appearing in later stages [7, 14]. Neutrophils and Th cells produce proinflammatory cytokines, such as TNF-α, IL-1, IL-23, and IL-17, which are of great importance in the pathogenesis of HS [7, 15]. Moreover, keratinocyte proliferation is caused by the linkage of IL-17 A to IL-17 receptor (IL-17R) A, IL-17RC, or IL-17RD [16]. In addition, there is evidence that the IL-23/IL-17 signaling pathway significantly impacts chronic inflammation in HS since IL-23 increases IL-17 production, a key cytokine associated with HS severity, by stimulating the development and differentiation of Th17 cells. Also, TNF-α raises the Th17/TREG ratio following Th17 polarization enhancement, which is a major contributor to disease development in lesion-involved tissues [17]. Likewise, elevated serum and lesional skin levels of TNF- α, IL1β, IL-10, and IL-17 in the HS subjects than healthy individuals propose these inflammatory markers as potential therapeutic targets as they affect different phases of HS pathology [18]. Notably, anti-TNF-α therapy correlated with a significant drop in Th17 cell number, confirming that TNF-α is critical in provoking IL-17 production in HS lesions [17]. Elevated TNF-a levels in serum and skin patients suffering from DCS along with the prevalent coexistence of DCS and HS as part of the FOT, support the relevance of TNF-a in the pathogenesis of DCS [5, 19]. These findings suggested that biologics can hold a promising effect on DCS management.

Moreover, the JAK/STAT pathways possess a crucial role in numerous inflammatory disorders [20]. Of note, JAKs are a kind of protein tyrosine kinases interacting with transmembrane type 1 and type 2 cytokine receptors to regulate cellular responses to different cytokines and growth factors, which plays a vital role in immune system function [21, 22]. Inhibiting the JAK-STAT pathway suppresses cytokine signaling, resulting in decreased serum inflammatory markers, such as C-reactive protein.

A variety of treatment modalities have been utilized in the management of DCS, including antibiotics, zinc sulfate, isotretinoin, corticosteroids, antiandrogens, laser therapy, aminolevulinic acid-photodynamic therapy, and different types of surgery [1]. There are, however, many prevailing issues associated with these therapies, including recurrence, relapses, limited effectiveness, and complications. Therefore, immunomodulators that suppress different parts of the inflammatory cascade in the HS can also be a potential treatment choice for DCS patients, given several similarities between the aforementioned disorders [5, 9]. Prior investigations indicated that TNF-α and IL inhibitors showed effectiveness in treating HS patients [23–26]. Additionally, previous evidence has demonstrated the potential efficacy of JAK inhibitors as novel options in the treatment of individuals with HS [27, 28]. Further, apremilast is a selective inhibitor of the enzyme phosphodiesterase 4 (PDE4), which reduces the serum level of inflammatory cytokines, such as TNF-α and different ILs [29]. Apremilast administration for HS patients has been associated with satisfactory results in HS cases [30]. A deeper understanding of the efficacy and safety of such immunomodulators in DCS holds great promise for patients who fail conventional therapies [31–35]. This systematic review aims to assess biologics’ clinical effectiveness and safety profile, including anti-ILs, TNF-α, blockers, and small molecule inhibitors, including JAK inhibitors and apremilast, in treating DCS.

## Methods

This systematic review was conducted based on Preferred Reporting Item for Systematic Reviews and Meta-Analysis (PRISMA) checklists [37]. These checklists are included in the S1 and S2 Tables.

### Search strategy

A systematic search was conducted through PubMed/Medline, Scopus, and Ovid Embase up to February 4 th, 2024. The S3 Table contains the complete list of search terms, including keywords and MeSH terms, and final systematic search results.

### Eligibility criteria and study selection

This systematic review included clinical trials, observational studies, case series, and case reports, with an available English full text. The eligible source populations were patients with no age limits who suffered from DCS and received TNF blockers, anti-ILs, PDE4 inhibitors, or JAK inhibitors. Non-English studies, review articles, guidelines, and experimental studies were excluded.

## Data extraction

Each study was extracted by two independent reviewers (RG and MF) through a data extraction sheet based on the following information: (I) Study Characteristics (author, year, design, sample size), (II) patients’ characteristics (mean age, gender distribution, past medical history and comorbidities, disease condition and duration, and previous treatments), and (III) outcomes (treatment efficacy, safety, and adverse events, follow-up).

### Risk of bias assessment

Two independent investigators (RG and MF) evaluated the articles’ methodological quality and bias risk by using the National Institutes of Health’s Quality Assessment Tool for Cohort and Cross-Sectional Studies [37] and Methodological quality and synthesis of case series and case reports Murad et al. [38] for case reports and case. S4 and S5 Tables illustrated the bias assessment results.

## Results

### Search results

**Fig. 1:**
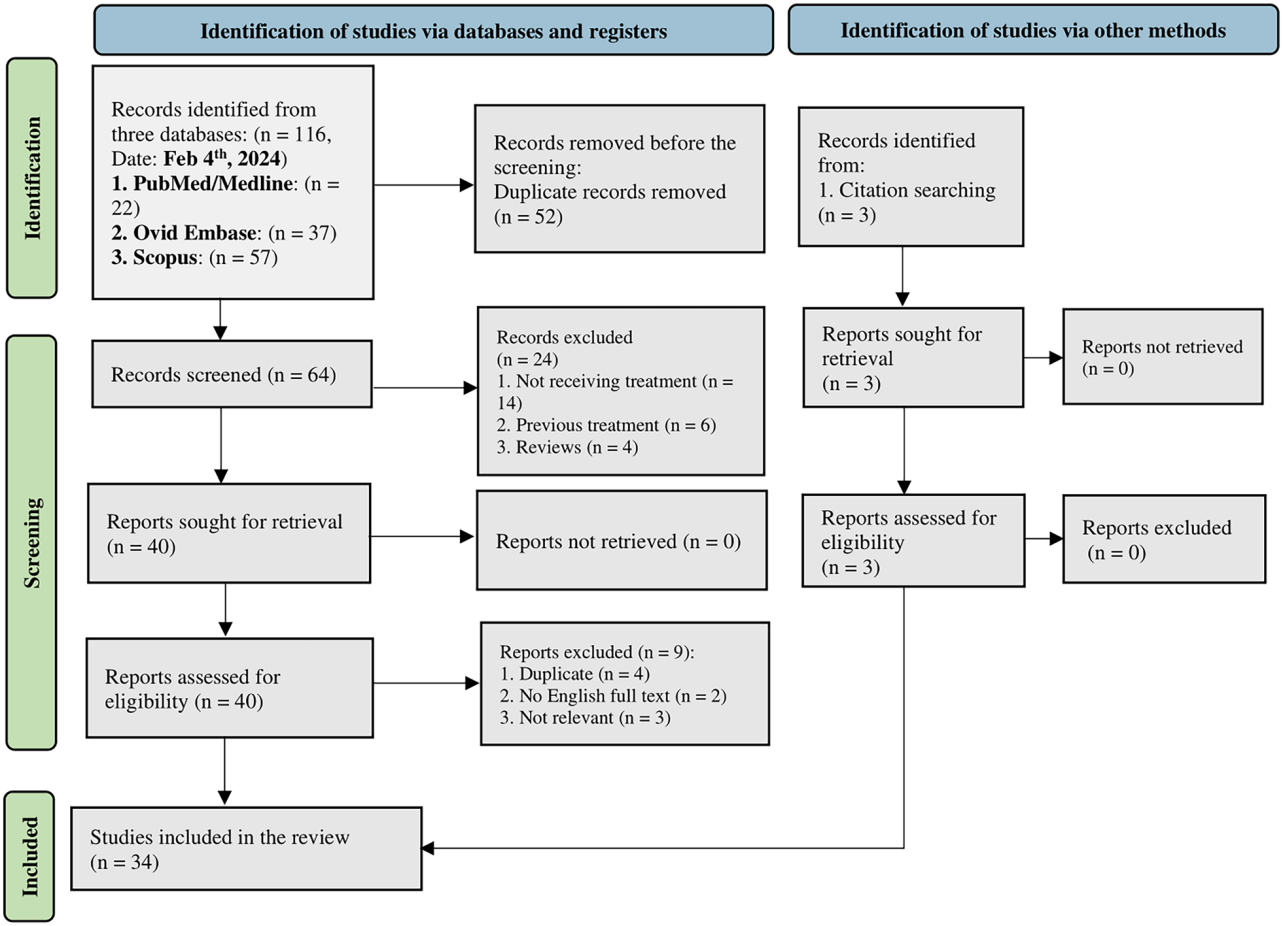
PRISMA 2020 flow diagram for new systematic reviews, which included searches of databases, registers, and other sources

The systematic literature search achieved 116 related studies, including 22 from PubMed, 57 from Embase, and 37 from Scopus, in the search up to February 4 th, 2024. A total of 64 articles were further screened based on title and abstract after removing the duplicates. After excluding further studies based on title and abstract screening, two independent investigators (NH and YG) evaluated the full texts of 40 articles, given the inclusion and exclusion criteria. In the ultimate evaluation, 31 articles were selected. Moreover, a citation search was conducted, and three studies were included. A total of 34 studies were included for data extraction. Figure 1 demonstrates the PRISMA flowchart of this systematic review.

### Characteristics of eligible studies

A total of 34 articles involving 81 patients with DCS who were treated with anti-TNF-α agents, IL blockers, and small molecule inhibitors, including JAK inhibitors and apremilast were included in the systematic review. The selected studies were as follows: four cohort studies, six case series, and 24 case reports. Although the gender of patients was not mentioned in two articles [39, 40], only three out of 73 patients (4.1%) were women based on the studies in which gender had been reported. Biologics studied for the treatment of DCS include adalimumab, infliximab, secukinumab, guselkumab, risankizumab, ustekinumab, cetulizumab pegol, and tildrakizumab. Moreover, small molecule inhibitors utilized for DCS patients include apremilast, upadacitinib, and baricitinib. All characteristics of eligible studies are summarized in Table 1.

**Table 1:** Characteristics of clinical studies on Tumor necrosis factor-α blockers, anti-interleukins, and small molecule inhibitors in the treatment of dissecting cellulitis of the scalp

Study ID (Author, year)	Study design	Sample size	Gender (M: male. F: Female; %)	Age (year ± SD)	Past medical history and comorbidities	Disease condition	Disease duration	Previous Treatment(s)	Treatment(s) of interest	Concurrent treatment(s)	Outcome measurement	Efficacy	Safety and adverse events	Follow-up
*Islam, 2024 *[34]	Case report	1	M	26	Obesity and atopic dermatitis	Enlarging, painful cysts, Elevated ESR, CRP, and IL-6	11 months	benzoyl peroxide 10%, sulfamethoxazole-trimethoprim 800–160 mg twice daily, oral and intralesional corticosteroids	Upadacitinib 15 mg twice daily	Topical antimicrobials, oral antibiotics, and corticosteroid injections	Clinical signs and symptoms	Substantial improvement in pain, pustular draining, bleeding, and quality of life, fewer pustules, smaller sinus tracts, and decreased inflammation with no visible drainage	No major side effects	NA
*Nagshabandi, 2023 *[33]	Case report	2	M	Pt1: 26 Pt2: 17	Pt1: type 2 diabetes, sleeve gastrectomy Pt2: NA	Pt1: multiple erythematous draining nodules with few pustules, and scarring alopecia patches Pt2: few erythematous nodules with hair regrowth mainly over the occipital scalp and crown a solitary alopecic patch over the right temporal area and occipital scalp, miniaturized hair with few yellowish globules on dermatoscopic evaluation	Pt1: 2 years Pt2: 5 years	Pt1: topical clindamycin 1% solution and oral doxycycline 100 mg Pt2: topical clindamycin solution, doxycycline 100 mg for 6 months, oral clindamycin 150 mg, oral rifampicin 300 mg, which increased to 600 mg, topical minoxidil 5%, oral zinc sulfate	Risankizumab SC injection	Pt1: NA Pt2: topical clindamycin 1% solution daily	Clinical signs and symptoms	Pt1: an improvement of roughly 70% of lesions by the fifth dose of risankizumab Pt2: clinical remission by the third dose of risankizumab	NA	NA
*Bernard, 2023 *[35]	Case report	1	M	54	Epilepsy, hyperlipidemia, diabetes mellitus, and hypertension	Several fluctuant, tender nodules throughout the posterior and vertex scalp	5 years	erythromycin and minocycline, trimethoprim-sulfamethoxazole 800/160 mg twice a day for 2 months, minocycline 100 mg twice daily for 3 months, dapsone 100 mg daily for 6 months, and isotretinoin 80 mg daily for 4 months, 1% clindamycin solution for 4 months, 2.5% selenium sulfide 2.5% wash for 1 year, 0.05% fluocinonide solution for 2 months, adalimumab 80 mg every 14 days for 5 months, intralesional corticosteroids, surgical treatment	Apremilast 30 mg twice daily	One in-clinic surgical deroofing procedure	Clinical signs and symptoms	Dramatic improvement of disease symptoms and reduction in flares	No adverse events	The patient remains on 30 mg of apremilast twice daily with sustained improvement of his DCS with additional procedures
*Yu, 2023 *[51]	Case report	1	M	15	BMI = 26.2, smoking, FH of skin appendage disorders	Several fluctuating pustules and prominent erythematous nodules on the scalp, interconnecting sinuses filled with malodorous pus, and extensive alopecia patches	10 months	Antibiotic treatment with oral minocycline (50 mg twice daily) for 3 months and oral clindamycin (0.15 g four times daily) for 1 month, operation to remove the abscess and drain the pus	Adalimumab and baricitinib 80 mg adalimumab on day 0, followed by 40 mg every 2 weeks, reduced to every 4 weeks after 4 months Baricitinib 4 mg daily started at month 4, which was reduced to 4 mg every 3 days 2 months later	Minocycline (50 mg twice daily), isotretinoin (30 mg daily), which was discontinued after 4 months, fenofibrate (200 mg daily) at month 4	Laboratory tests, clinical signs and symptoms, hair regrowth	Improvement of the scalp with a drop in the number of fresh pustules and less drainage, alopecia patches became scattered, tenderness subsided, hair regrowth, control of inflammation, leukocyte and neutrophil counts altered from 12.7 × 10^9 and 9.1 × 10^9 to 6.9 × 10^9/L, 3.5 × 10^9/L respectively, and lymphocyte changed from19.8% to 39.1%, CRP concentration dropped from 3.38 mg/L. to 1.73	Elevated triglycerides and cholesterol levels (related to adalimumab)	NA
*Alzahrani, 2023 *[43]	Cohort study	26	M: 96%	24 ± 10	BMI > 30 (42%), cigarette smoking (54%), cannabis consumption (11%)	NA	NA	Systemic antibiotics (92%), isotretinoin (65%) and oral corticosteroids (11%)	Infliximab and adalimumab (anti-TNFα) For 19 months Adalimumab (*n* = 5, 19.2%) 40 mg every 2 weeks and infliximab (*n* = 21, 80.8%) starting at a dose of 5 mg kg^−1 every 4, 6 or 8 weeks, increased to 7.5 mg kg^−1 in six patients, and to 10 mg kg^−1 in four patients	NA	PGA, number of inflammatory nodules and abscesses, DLQI, PSI	The median PGA score decreased,The median number of inflammatory nodules and abscesses decreased, The median DLQI and the NRS score for pain severity decreased, The median PSI was 7 out of 10	Optic neuritis (*n* = 1, after 3 months of treatment) and hepatic cytolysis (*n* = 1 after the ninth perfusion)	2 patients were in remission, 3 demonstrated moderate efficacy and 1 was lost to follow-up
*Almuhanna, 2023 *[62]	Case report	1	F	33	Pregnancy (12 weeks)	Diffuse, boggy, hyperkeratotic, and atrophic plaques with overlying crust and scattered alopecic patches on scalp	5 years	Topical and systemic antibiotics, systemic corticosteroids, isotretinoin	Certolizumab pegol SC loading dose: 400 mg at weeks 0, 2, and 4, 200 mg every other week	Cephalexin 500 mg, 2 times a day for 10 days	Clinical signs and symptoms	70% improvement of lesions, with less pruritus, tenderness, erythema, purulent discharge, crustation, and reduction in the frequency of new lesions (after 4 months)	Tolerable with no adverse reactions	Sustained treatment response (after 8 months, 4 weeks postpartum), remained on certolizumab 200 mg every 2 weeks
*Koike, 2022 *[20]	Case report	1	M	21	NA	Elastic, soft, SC walnut-sized nodules with purulent secretion and geographic hair loss in the occipital region, soft tissue inflammation of the scalp, An IHS4 score of 65 points, elevated white blood cell counts and serum CRP	6 years	Oral minocycline, surgical treatment	Adalimumab An initial dose of 160 mg, tapering to 80 mg and then to 40 mg every 2 weeks	NA	IHS4, clinical signs and symptoms	A decrease in IHS4 from 22 to 7 points, after 1 month of treatment and to 3 points after 4 months Flattening of the scalp lesion and hair regrowth Deacreased serum level of IL-1RA, IL-1b, MCP-3, MCP-1, MIP-1a, IL-8 and FGF-2 after 3 months of adalimumab treatment	NA	NA
*Gamissans, 2022 *[42]	Cohort study	3 (a total of 14 patients, 3 receiving anti-TNFα)	M	39.6 ± 9.8	Median BMI = 28.5 kg/m2, smoking (64%, *n* = 9), HS (86%, n = 12)	DCS stage II or III (79%, *n* = 11) lesions predominated on the vertex (100%, *n* = 14) occipital area (50%, *n* = 7)	NA	NA	Adalimumab and Infliximab (anti-TNFα) For 9.33 ± 3.77 months, 66.6% (*n* = 2) adalimumab 80 mg/2 weeks, infliximab 33.3% (*n* = 1) 0.5 mg/kg/month	Topical or oral antibiotics or intralesional steroid injection	Hair regrowth and absence of a bald area	Complete response (66.6%, *n* = 2) and partial response (33.3%, *n* = 1)	Infusion reaction leading to treatment withdrawal (33%, *n* = 1)	0% recurrence rate
*Babalola, 2022 *[63]	Case report	1	M	65	Congestive heart failure (ejection fraction of 45%), coronary artery disease, hyperlipidemia, hypertension, and non-alcoholic fatty liver disease	Recurrent itchy bumps, persistent draining nodules on the scalp, multiple tenders, crusted lesions with suppurative drainage on the parietal and occipital scalp, severe mucocutaneous dryness	13 years	Topical clindamycin, doxycycline 100 mg BID, intermittent intralesional triamcinolone, chlorhexidine gluconate, fluocinonide, and isotretinoin 30 mg BID	Risankizumab 150 mg every 12 weeks	NA	Clinical signs and symptoms	Well response to treatment	NA	NA
*Awad, 2022 *[64]	Case report	1	M	28	HS, AC	Several large, fluctuant, tender nodules on the scalp with overlying alopecia	NA	Isotretinoin 20 mg, erythromycin 500 mg, and intralesional triamcinolone injections	Tildrakizumab (IL-23 inhibitor) For 8 weeks, 2 doses of SC injection 4 weeks apart	NA	Clinical signs and symptoms, number and severity of pustules and scalp tenderness, hair regrowth	Significant reduction in the number and severity of pustules and alleviated scalp tenderness along with hair regrowth in the areas of alopecia	NA	NA
*Sanchez-Diaz, 2021 *[48]	Case series	2 (a total of 8 patients, 2 with DCS receiving anti-TNFα)	M	Pt1: 48 Pt2: 45	Pt1: BMI 39.4, HS Pt2: BMI 48.97, smoking	Pustules, nodules, abscesses, scars, and fistulas on the scalp	Pt1: 480 weeks Pt2: 432 weeks	Pt1: doxycycline, clindamycin-rifampicin, isotretinoin, photodynamic therapy, adalimumab Pt2: doxycycline, rifampicin, isotretinoin, acitretin	Pt1: infliximab (anti-TNFα) 5 mg/kg every 8 weeks Pt2: adalimumab (anti-TNFα) 80 mg every 2 weeks	Pt1: dapsone (100 mg/day), ertapenem (1 g IM/24 h) Pt2: NA	NRS pain, NRS pruritis, NRS suppuration, Hurley, IHS4, AN, HiSCR	HiSCR achieved. a significant decrease in NRS pain, NRS pruritis, NRS suppuration, Hurley, IHS4, and AN	NA	Pt1: satisfactory response and reached HiSCR in follow-up after 24 weeks Pt2: satisfactory response and reached HiSCR in follow-up after 32 weeks
*Spiers, 2021* [50]	Case report	1	M	34	NCA, ankylosing spondylitis, cigarette smoking	Severe dissecting scalp cellulitis, type VI skin, soft boggy swellings and abscesses affecting the scalp, worse in the occipital region (the largest: 6 × 3 cm), new lesions and interconnected sinuses in the parietal and occipital area, all discharging pus	NA	A three-month course of rifampicin 300 mg and clindamycin 300 mg, both twice daily, and a 4-month course of oral isotretinoin, 4 staged procedures of incision, drainage, and excision of scalp lesions, with the wounds being packed and left to heal by secondary intention	Adalimumab 40 mg fortnightly from 14 months before the surgical procedure to 8 months after it	Surgical management (two further staged procedures: incision, drainage, and excision of large areas of scalp under general anesthetic)	DLQI, clinical signs and symptoms	Self-report of 10–15% improvement (while on adalimumab before surgery), DLQ1 dropped from 14/30 to 2/30, complete healing of the scalp without discharge (after surgery)	NA	No further discharge or episodes in one-year follow-up
*Minakawa, 2021 *[52]	Case report	1	M	30	Smoking (10 years), BMI = 30.49, HS	Multiple soft subcutaneous nodules with oozing, purulent secretions from fistulas all over scalp and face, patchy hair loss, massive diffuse lymphocytic infiltration in the dermis on biopsy, perivascular and perifollicular areas, T cell infiltration was visualized using the pan-T cell marker CD3, but TNF- was negative	12 years	Minocycline 200 mg/day for 4 years, Nadifloxacin, Benzoyl peroxide, and Clindamycin phosphate	Adalimumab SC injections for 1 month	NA	Pain and severity of secretions, WBC, CRP, sialylated carbohydrate antigen KL-6, DLQI	Decrease in pain, cessation of purulent secretions, normalized WBC count, CRP levels, and KL-6 levels, improvement of DLQI after 18 months (2 to 0)	NA	NA
*Kurokawa 2021* [58]	Case report	1	M	18	Insomnia, HS (Hurley stage I), NCA	Severe multiple painful, itchy hemorrhagic ulcerations, nodules, hypertrophic scar, and alopecia of the occipital area	6 years	Oral faropenem 600 mg/day and Saireito (Japanese herb) 8.1 g/day for 4 weeks	Adalimumab 160 mg of adalimumab on day 1, and subsequently, 80 mg every other week for 1 month	NA	Clinical signs and symptoms, hair regrowth, stabilization of the co-existing diseases	Reepithelialization of hemorrhagic ulceration, resulting in scar formation, a decrease in severe pain and itching, resolved insomnia, absence of the nodules on the occipital area, improvement of alopecia leading to hair growth, improvement of NCA on the face and nodules on the buttocks, control of HS (IHS4 reduced from 3 points to 0)	NA	NA
*Frechet, 2021* [45]	Case series	9	M: 88.88%	33 ± 13	Obesity (44%), active smoking (44%). AC (66%), HS (66%), pilodinal sinus (11%), psoriasis (11%) , HLA B27-negative spondylitis (11%)	NA	NA	Systemic antibiotics (78%), isotretinoin (67%), methotrexate (11%), disulone (11%), thalidomide (11%), oral corticosteroids (11%), apremilast (11%), canakinumab (11%), and tocilizumab (11%)	Infliximab and adalimumab With a mean duration of 17 ± 16 months Infliximab (89%) 5 mg/kg at weeks 0, 2, 6, and, every 8 weeks in 5 (63%) patients, and every 6 weeks in 3 (37%) patients, dosing was increased to 7.5 mg/kg in 1 patient Adalimumab (11%) (40 mg every two weeks) without a washout period	Isotretinoin (33%), oral corticosteroids (33%), doxycycline (33%), and methotrexate (11%)	PGA, number of inflammatory nodules and abscesses, DLQI, treatment satisfaction index	The mean PGA score decreased from 4 ± 1 to 2 ± 1, the mean number of inflammatory nodules dropped from 9 ± 3 to 3 ± 4 (67% reduction), the mean number of abscesses decreased in 7/8 patients (89%) from 1.7 ± 1.06 to 0.2 ± 0.7 (78% reduction), the mean DLQI reduced from 27 ± 4 to 12 ± 8 (45% improvement), the mean treatment satisfaction index was 6.6 ± 1.6 out of 10, An increase in CPR and hyperleukocytosis persisted in one patient (75% reduction)	Retrobulbar optic neuritis leading to discontinuation of infliximab (n = 1)	Continuing the treatment for 17 ± 16 months
*De Bedout, 2021* [65]	Case report	1	M	63	Acne vulgaris	Scarring alopecia with tender, fluctuant, purulent nodules	4 years	Doxycycline, trimethoprim-sulfamethoxazole, clindamycin, rifampin, and adalimumab, oral dapsone 12.5 mg daily with a gradual increase to 50 mg daily and concomitant intralesional triamcinolone 10 mg/cc	Secukinumab (IL-17 inhibitor) 150 mg weekly for 6 weeks (patient mistakenly took an extra loading dose) then monthly for 2 months A total of 8 injections of 150 mg over three months	Dapsone 50 mg daily	Clinical signs and symptoms	Complete cessation of drainage and pain, regression of nodules	Eczematous reaction	The patient remained in remission at one-year follow-up
*Alsantail, 2021* [32]	Case report	1	M	38	NA	Inflammatory, boggy, fluctuant nodules on the upper occiput with recurrent foul-smelling discharge. scalp punch biopsy: epidermal hyperkeratosis, neutrophilic infiltrate of the hair follicles and deep dermis, and focal areas with multinucleated giant cells and histocytes (foreign body giant cell reaction)	5 yearts	Several topical and systemic antibiotics (clindamycin, doxycycline, and amoxicillin/clavulanic acid), isotretinoin for 17 months, with dose escalation to 1 mg/kg (80 mg/day)	Adalimumab 80 mg on day 0, then 40 mg on day 7, and 40 mg weekly thereafter	NA	Clinical signs and symptoms, hair regrowth	Excellent response after 1 month, less pain, no more discharge, decreased swelling, and areas of hair regrowth after 2 months	NA	The patient continues to receive 40 mg of adalimumab weekly
*Philips, 2020 *[40]	Case series	1 (a total of 28 patients, 1 receiving ustekinumab)	NA	NA	IBD	NA	NA	Anti-TNFα	Ustekinumab (IL-12/23 inhibitor)	Topical therapies	Clinical signs and symptoms	No response to ustekinumab	NA	NA
*Muzumdar, 2020 *[66]	Case report	1	M	NA	HS, folliculitis, AC, and pyoderma gangrenosum	Multiple, painful, and tender fluctuant 1—2 cm nodules diffusely over the scalp, associated with patchy scarring alopecia	4 years	Methotrexate, minocycline, adalimumab 40 mg SC every week, hydroxychloroquine 200 mg BID, doxycycline 100 mg BID, prednisone 10 mg once daily, and intermittent topical clobetasol cream	Guselkumab 100 mg SC 4 weeks apart for the first two doses, then every 8 weeks thereafter for 6 months	NA	Clinical signs and symptoms	Near-complete resolution of the scalp lesions associated with the resolution of all symptoms	Tolerable with no side effects	NA
*Maxon, 2020* [53]	Case report	1	M	37	Extensive cystic acne	Bogginess, fluctuance, large, firm, skin-colored to erythematous nodules with overlying patches of scarring alopecia on the occipital scalp, several smaller erythematous nodules on the anterior frontal scalp	13 years	Serial intralesional corticosteroid injections, excision of scalp lesions, oral isotretinoin, intermittent oral antibiotics	Adalimumab 40 mg once weekly	NA	Clinical signs and symptoms, hair regrowth	Significant clinical improvement after 2 months, notable hair regrowth and reduction in bogginess and tenderness of the scalp after 6 months	NA	He continued therapy with adalimumab, but after 2 years of treatment, clinical improvement plateaued. He was subsequently placed back on the oral retinoid acitretin with additional improvement
*Cautela, 2020 *[39]	Case series	7	NA	NA	HS	NA	NA	NA	Adalimumab 160 mg at week 0, followed by 80 mg at week 2, then 40 mg from week 4 and thereafter every week	NA	Clinical signs and symptoms	Rapid reduction in clinical signs of inflammation and burden of disease	NA	NA
*Takahashi, 2019 *[5]	Case report	1	M	19	BMI = 31.1, HS	Multiple, soft subcutaneous nodules with oozing, purulent secretion from fistulas, patchy hair loss on the scalp, irregular skin surface caused by fistulas and scars resembling a so-called cutis verticis gyrata, multiple abscesses and fistulas reaching as deep as the skull bone in magnetic resonance imaging	5 years	Clarithromycin and zinc supplementation for 3 months	Adalimumab SC injection of 80 mg on day 0, followed by 40 mg every other week, increased from 40 to 80 mg every other week at 3 months	NA	Clinical signs and symptoms, hair regrowth, stabilization of the co-existing diseases, laboratory tests	Improvement of pain and purulent secretion after 1 month, partial hair regrowth and diminished inflammatory skin lesions except for post-inflammatory hyperpigmentation and hypertrophic scars in axillae after 3 months, achieved HS clinical response, normalization of WBC counts and CRP level	NA	Continuing over 9 months with favorable response
*Syed, 2018 *[61]	Case report	1	M	31	Peptic ulcer disease status post partial gastrectomy, Crohn’s disease	Multiple erythematous interconnecting plaques, some boggy with dried yellow crust on the frontal, parietal, and occipital scalp with scant purulent drainage	2 years	Antibiotic treatment	Infliximab (anti-TNFα)	Steroids	Clinical signs and symptoms, hair regrowth, stabilization of the co-existing diseases,	Complete remission of the skin disease and gastrointestinal symptoms	NA	NA
*Sjerobabski Masnec, 2018 *[54]	Case report	1	M	26	BMI: 35.8, smoking, HS (Hurley stage II), facial acne	Progressive patchy hair loss overlying inflammatory papules, pustules, yellow crusts, and tender, fluctuant, suppurative nodules (frontal scalp), several confluent conglomerates nodules, which discharged purulent secretion when pressed, fistulae, interconnecting sinuses, swelling of regional lymph nodes	NA	Isotretinoin at a dose of 0.64 mg/kg over 10 months, multiple antibiotics	Adalimumab 80 mg on days 0, 1, and 14 followed by 40 mg on day 28 and every week there- after	NA	Clinical signs and symptoms, stabilization of the co-existing diseases, DLQI	Significant improvement of all symptoms, reduced secretion, pain, and inflammatory changes on the scalp, absence of new nodules and sinus tracts in the bilateral axilla, inguinum, and pubic region, as well as clearing of facial acne, DLQI dropped significantly from 27 to 1	Tolerable with no adverse reactions	Continuing adalimumab 40 mg injections every week over 9 months with desireable response and tolerability
*Mansouri, 2016 *[55]	Case report	2	M	Pt1: 48 Pt2: 27	Pt1: HS, abnormal liver function tests (ALT twice the upper limit, GGT 37 times the upper limit of normal) Pt2: NA	Pt1: malodorous, tender lesions on the scalp, perifollicular scaling, pustule scarring alopecia Pt2: inflamed scalp, suppuration, and inflammatory papules, numerous perifollicular pustules, tender plaques with foul-smelling discharge, scarring alopecia on the scalp	Pt1: 20 years Pt2: 4 years	Pt1: multiple antibiotics, zinc sulfate, dapsone, isotretinoin, systemic corticosteroids, surgical excision and drainage Pt2: topical and systemic corticosteroids, antibiotics including dapsone, and isotretinoin	Pt1: adalimumab 80 mg on day 0, followed by 40 mg on day 7 and 40 mg every other week thereafter Pt2: infliximab 5 mg kg 1 at weeks 0, 2 and 6, followed by 8-week intervals for 20 months	NA	Clinical signs and symptoms, DLQI	Pt1: reductions in inflammation and pain after 1 month, improvement in liver enzymes (ALT and alkaline phosphatase within the normal range), DLQI reduced significantly from 21 to 10 after 5 months, with marked reduction in discharge Pt2: reduction of symptoms, inflammation and odour within 3 months of treatment, DLQI reduced from 18 to 6 after 12 months	NA	NA
*Badaoui, 2016 *[41]	Cohort study	1 (a total of 51 patients, 1 receiving infliximab)	M: 98%	NA	HS (*n* = 6, 12%), AC (*n* = 8, 16%), both AC and HS (*n* = 2.4%)	Subcutaneous nodules and abscesses located on the vertex (*n* = 25; 49%), diffuse over the entire scalp (*n* = 4, 9.8%) Mild (2%), moderate (61%), and severe DCS (25%) A traumatic trigger (*n* = 5): hair shaving; neurosurgery for epilepsy or after wearing a helmet Nodules: painful (*n* = 13, 25%) and itchy (*n* = 4, 8%) The pattern of disease progression: chronic with progressive onset of lesions (*n* = 44; 86%), acute (*n* = 7, 14%)	34.3 (4–12) months	NA	Infliximab	NA	Clinical signs and symptoms	No improvement	NA	11.2 months of follow-up
*Sand, 2015 *[44]	Cohort study	2	M	NA	NA	Severe DCS	NA	Isotretinoin, dapsone, triamcinolone	Adalimumab 40 mg twice monthly	NA	Clinical signs and symptoms	1/2 (50%) response rate, an elderly man obtained total clearance of the disease within 3 months of therapy, whereas a young male patient did not respond to 6 months of therapy	No adverse events	NA
*Martin-Garcia, 2015 *[56]	Case report	1	M	30	NA	Scattered tender fluctuant nodules on the scalp, overlying alopecia	15 years	Intralesional triamcinolone, doxycycline, ciprofloxacin, isotretinoin	Adalimumab 80 mg on day 0, 40 mg on day 7, 40 mg every other week thereafter	NA	Clinical signs and symptoms	A significant decrease in pain and swelling of the lesions after 1 month, which progressively improved, complete clearance of inflammatory lesions after 7 months	No adverse reaction	Continuing the treatment over 2 years
*Prastou, 2014 *[49]	Case report	1	M	49	Recurrent generalized folliculitis and furunculosis, microcytic anemia attributed to the beta-thalassemia trait, chronic abnormal cholestatic liver function tests, and hypertension	NA	NA	Oral antibiotics, intralesional and oral steroids, isotretinoin, dapsone, intermittent courses of ciprofloxacin over the previous 6 months, and bendroflumethiazide	Adalimumab 40 mg subcutaneously every fortnight	Ciprofloxacin and bendroflumethiazide	DLQI, clinical signs and symptoms	Improvement in scalp inflammation and discharge and reduction in DLQI from 21 to 10 at month 5	A tender lump on the right lower leg new tender panniculitis lesions on the lower limbs (after 2 months)	NA
*Lim, 2013 *[47]	Case series	1 (a total of 5 patients, 1 with DCS receiving anti-TNFα)	M	29	Sycoses barbae, AC, ankylosing spondylitis peripheral (LE) Achilles tendonitis	A few abscesses and multiple, crusted tender nodules with patchy alopecia over the vertex of the scalp	Pt1: 20 years	Pt1: rifampin and clindamycin	Adalimumab 40 mg SC every other week	Transretinoin cream and fluocinonide 0.05% cream, NSAID	Clinical signs and symptoms, stabilization of the co-existing diseases	Marked symptomatic improvement, resolution of Achilles tendonitis and knee synovitis, and his BASDAI score decreased from 5.2 to 1.2, asymptomatic and free of any skin lesions	NA	Asymptomatic, remained on adalimumab every 2 weeks
*Wollina, 2012 *[59]	Case report	1	M	30	Smoking, type 2 diabetes mellitus	inflammation, painful nodules on the scalp, malodorous discharge from enlarged pores, scarring alopecia and keloid-like scars, painful and swollen nuchal and submandibular lymph nodes	1 year	Rifampicin, isotretinoin, prednisolone, ibuprofen, metamizole, amitriptyline, minor surgery	Infliximab IV 5 mg/kg body weight at weeks 0, 2, and 6	Surgical management	Clinical signs and symptoms, CRP,	Significant reduction in inflammation, secretion, pain, and nodules, decreased CRP dropped (from 19.1 mg/L to 2.6 mg/L), complete disappearance of lymph node swelling, mood improvement	Psoriasiform exanthema induced by TNF-α inhibitor, which was completely managed by topical prednicarbate ointment	Nearly complete remission at 3 month follow-up
*Navarini, 2010 *[46]	Case series	3	M	Pt1: 27 Pt2: 29 Pt3: 30	Pt1: NA Pt2: NA Pt3: HS	Boggy and fluctuant infiltrates with purulent secretion Pt1: pronounced inflammatory infiltrate, intermediate fibrosis and cicatrization Pt2: detectable inflammatory infiltrate, no fibrosis, and cicatrization Pt3: detectable inflammatory infiltrate, detectable fibrosis and cicatrization	Pt1: 1 year Pt2: 4 years Pt3: 7 years	Pt1: antibiotics Pt2: antibiotics, tetracyclines, isotretinoin Pt3: antibiotics, tetracyclines, levofloxacin, isotretinoin	Adalimumab At a dose of 80 mg SC followed by a dose of 40 mg 1 week later and an additional 40 mg every second week	NA	SDAS, inflammatory infiltrate, fibrosis and cicatrization, biopsy	Pt1: SDAS dropped from 5 to 2, reduction in inflammatory infiltrate, amelioration of clinical symptoms, pronounced fibrosis, and cicatrization Pt2: SDAS dropped from 8 to 2, remaining preexisting pathologic residual structures such as subcutaneous sinus tracts, amelioration of clinical symptoms, absence of fibrosis, and cicatrization Pt3: SDAS dropped from 7 to 2, reduction in inflammatory infiltrate, amelioration of clinical symptoms, detectable fibrosis, and cicatrization	NA	Restarting adalimumab in Pt3 since disease activity returned within 4 weeks
*Sukhatme, 2009 *[57]	Case report	1	M	39	NA	Painful, tender fluctuant mass on posterior scalp	6 years	Multiple courses of antibiotics and intralesional triamcinolone injections, excision, oral isotretinoin	Adalimumab (anti-TNFα) Two 40-mg SC injections for the first week, 40 mg for the second week, and then 40 mg every other week	NA	Clinical signs and symptoms, hair regrowth	After 2-months there were two slightly boggy flesh-colored nodules with hair growth with no erythema or purulent drainage	NA	At the 5-month follow-up, his lesions had cleared, and his hair was growing back normally
*Brandt, 2008 *[60]	Case report	1	M	24	NA	Pustules, tender nodules and sinus tracts on the scalp, scarring alopecia Dermal sclerosis and fibrosis	4 years	Dapsone, doxycycline, ciprofloxacin and isotretinoin	Infliximab 5 mg/kg infused at 8-week intervals for 12 months, for a total of six infusions	NA	Hair regrowth, clinical signs and symptoms	Excellent response, with hair beginning to regrowth after the second infusion, continued	No adverse effects	One year after discontinuing infliximab, the hair regrowth was maintained with no signs of residual inflammation or relapse of the disease

AC: Acne conglobate; ALT: Alanine aminotransferase; AN: Sum of abscesses and nodules; BASDAI: Bath ankylosing spondylitis disease activity index; BID: twice a day; BMI = Body mass index; CRP: C-reactive protein; DLQI: Dermatology life quality index; ESR: Erythrocyte sedimentation rate; FH: Family history; FGF: Fibroblast Growth Factor; GGT: Gamma-glutamyltransferase; HiSCR: Hidradenitis suppurativa clinical response; HS: Hidradenitis suppurativa; IHS4: International hidradenitis suppurativa severity score system; IHS4: International hidradenitis suppurativa severity; IL: interleukin; IV: Intravenous; JAK: Janus kinase; L: Liter; LE: lower extremity; MCP: Monocyte Chemoattractant Protein; MIP: Macrophage Inflammatory Protein-1 Alpha; NA: Not attributable; NCA: Nodulocystic acne; NRS: Numeric rating system; PCAS: Perifolliculitis capitis abscedens et suffodiens; PGA: Physician’s Global assessment scale; PSI: Patient satisfaction index; Pt: patient; SC: Subcutaneous; SDAS: Subjective disease activity score; TNF-α: Tumor necrosis factor-α; WBC: White blood cell

### Biologics

#### Tumor necrosis factor-α blockers (TNF-α blockers)

Anti-TNF-α agents, including adalimumab, infliximab, and certolizumab pegol, were administered to 71 subjects suffering from DCS in four cohort studies, four case series, and 18 case reports. Adalimumab was the most immunomodulator drug studied, with the dose ranging from 40 to 160 mg every 1–2 weeks. The infliximab dosing interval was 5 mg/kg every 4–8 weeks. Moreover, HS and AC were reported as a comorbidity in 22.3% (15/67) and 11.94% (8/67) of subjects. However, in two cohort studies that evaluated the efficacy of various treatments in DCS, the association of HS with anti-TNF-treated HS and AC patients was not mentioned [41, 42].

A retrospective study conducted by Gamissans et al. [42] investigated adalimumab and infliximab as two potential agents for treating cases resistant to conventional therapies, such as isotretinoin, dapsone, and surgery. Based on this study, 14 DCS patients, including 11 patients with DCS stage II and III, were investigated. Of 14 patients, only three received anti-TNF-α: two were treated with adalimumab, and infliximab was administered to one patient. As a result, a complete response was observed in two patients, and one patient experienced a partial response. Nonetheless, treatment withdrawal following infusion reaction was reported in one patient.

Another retrospective cohort study designed by Alzahrani et al. [43] studied 26 patients who were ineffectively treated by systemic antibiotics (92%), isotretinoin (65%), and oral corticosteroids (11%). In 24 cases, TNF-α blockers were started as the third-line treatment. Adalimumab and infliximab were administered in five and 21 patients, respectively. After 19 months, a remarkable decrease in the median number of inflammatory nodules and abscesses, physician’s global assessment (PGA), dermatology life quality index (DLQI), and severity of pain were observed. Furthermore, the median patient satisfaction index was claimed to be seven out of ten. In this study, however, anti-TNFs were discontinued in eight patients for several reasons. Two of these cases developed severe adverse events, including optic neuritis and hepatic cytolysis. Among the individuals who discontinued the treatment, two were in remission, three showed moderate efficacy, and one was lost to follow-up.

Badaoui et al. [41] investigated 51 DCS patients in a retrospective study, one of whom received infliximab. The patient did not show any significant improvement in 11.2 months of follow-up. Moreover, two subjects with DCS were assessed in a cohort study conducted by Sand et al. [44] that aimed to use the off-label TNF-α inhibitors in multiple dermatological conditions. After the failure of isotretinoin, dapsone, and triamcinolone, patients decided to receive adalimumab. Within three months of treatment, one patient achieved a complete clearance of the disease, while the other one did not demonstrate a considerable response despite six months of therapy. During the treatment period, these patients reported no adverse events.

In a case series employed by Frechet et al. [45], adalimumab and infliximab were given to 9 patients who were unresponsive to several treatments. Following the treatment, a notable reduction in PGA and DLQI was detected, as well as the number of inflammatory nodules and abscesses. Additionally, the mean treatment satisfaction index was found to be 6.6 ± 1.6 out of ten. Regarding adverse events, retrobulbar optic neuritis occurred in a patient receiving infliximab, leading to treatment discontinuation. Furthermore, Navarini et al. [46] assessed the efficacy of adalimumab in three DCS patients who had been treated with antibiotics and isotretinoin unsuccessfully. Ultimately, the patients reached a prominent improvement in clinical symptoms within eight weeks and a significant reduction in subjective symptoms after three months. An episode of relapse was observed in one patient after stopping the medication. Moreover, adalimumab administration led to a remarkable reduction in clinical inflammation and burden of disease in seven cases with DCS and severe HS [39]. In addition, Lim et al. [47] assessed five patients with spondylitis and Achilles tendonitis in another case series. Only one patient received adalimumab for his concurrent DCS and obtained sustained improvement in DCS and stabilization of the co-existing diseases without any complication. Moreover, Sanchez-Diaz et al. [48] utilized adalimumab and infliximab to treat two patients with DCS. In both cases, a marked clinical response for hidradenitis suppurativa clinical response was achieved, along with a significant reduction in disease indicators such as nodulocystic acne pain, pruritis, suppuration, Hurley stage of disease, International hidradenitis suppurativa severity, and abscesses and nodules count. Moreover, this favorable response persisted for up to 24 weeks of follow-up in the patient treated with infliximab and extended to 32 weeks of follow-up in the patient treated with adalimumab.

A total of 18 case reports evaluated the efficacy of adalimumab, infliximab, and certolizumab pegol in 16 peers with DCS. Thirteen cases received adalimumab for their condition [5, 20, 32, 49–58]. Signs and symptoms completely resolved after adalimumab administration in 13 patients. Only one patient did not respond to the treatment properly and underwent surgical management [50]. Nevertheless, two patients exhibited adverse events, such as an elevation in triglyceride and total cholesterol serum levels and a tender lump and panniculitis lesions located on the lower limb [49, 50].

Infliximab was administrated in four patients due to an unsuccessful treatment by conventional medications [55, 59–61]. All patients achieved favorable outcomes following infliximab. However, one patient developed a psoriasiform exanthema as a side effect [59].

Among all cases, only one patient was treated with certolizumab pegol [62]. Certolizumab pegol, in conjunction with cephalexin, was given to a female patient at week 12 of her pregnancy. After four months, the patient experienced a reduction in purulent discharge, tenderness, and erythema. Also, certolizumab pegol was safe and tolerable during pregnancy, as no adverse reaction was observed.

#### Anti-interleukins (Anti-ILs)

Anti-ILs had been administered in a total of seven DCS patients in five case reports [33, 40, 63–66]. These biologics include risankizumab, which targets IL-23 A; tildrakizumab, which is designed to block IL-23; secukinumab, an anti-IL-17 A; guselkumab, a monoclonal antibody against IL-23, and ustekinumab that acts against both IL-12 and IL-23. 28.5% (2/7) of subjects suffered from concomitant HS and AC [64, 66]. The dose range of IL inhibitors was as follows: risankizumab: 150 mg every 12 weeks; secukinumab: an anti-IL-17 A; and guselkumab: 100 mg every four weeks for the first two doses and then every eight weeks. The dose range of tildrakizumab was not reported [64].

In a study performed by Phillips et al. [40], ustekinumab did not lead to a favorable response in a DCS patient suffering from inflammatory bowel disease. In contrast to ustekinumab, other anti-ILs demonstrated a significant improvement in signs and symptoms of patients with other comorbidities who were resistant to previous therapeutic options. Muzumdar et al. [66] reported the use of guselkumab in a patient suffering from DCS, HS, folliculitis, AC, and pyoderma gangrenosum who was refractory to all conventional therapies and adalimumab. In this patient, switching adalimumab to guselkumab led to near‑complete healing of the scalp lesions and resolution of all symptoms. Furthermore, administration of tildrakizumab in an individual with a history of concurrent DCS, HS, and AC resulted in hair regrowth as well as a significant reduction in scalp tenderness and the number and severity of pustules [64]. Risankizumab also demonstrated a desirable efficacy and safety profile in three subjects with DCS [33, 63]. In addition, treatment with secukinumab resulted in complete cessation of drainage and pain and regression of nodules in a patient with DCS [65]. Despite eczematous reactions and treatment discontinuation due to lack of insurance coverage, the subject remained in remission at a one-year follow-up. However, it is important to note that while the effectiveness of these treatments in managing DCS has been documented, no data is available on the evolution of concurrent Pyoderma Gangrenosum, HS, and AC in these cases.

### Small molecule inhibitors

#### Janus kinase inhibitors (JAK inhibitors)

JAK inhibitors, including upadacitinib (JAK1 inhibitor) and baricitinib (JAK1/2 inhibitor), have been applied in two DCS patients [34, 51]. Upadaticinib administration in a 26 year-old male with DCS who failed previous treatment resulted in significant improvement in the pain, pustular draining, and bleeding with no major side effects [34]. Yu et al. [51] described a case where a combination of the JAK inhibitor baricitinib and the TNF blocker adalimumab was used to treat a 15 year-old patient with DCS. This treatment regimen led to improvements in scalp condition, control of inflammation, disappearance of alopecic patches, and hair regrowth. Notably, while the patient did not experience any adverse reactions directly related to baricitinib, an increase in triglyceride and cholesterol levels was reported. It is important to note that dyslipidemia can be associated with both adalimumab and baricitinib, and attributing it solely to adalimumab may not fully reflect the contributions of baricitinib. As there are limited number of patients reported and the nature of the available literature (primarily case reports), further studies with larger patient populations and controlled settings are needed to better understand the effectiveness and safety profile of JAK inhibitors for this condition.

#### Apremilast (Phosphodiesterase-4 inhibitor)

To date, apremilast has been investigated in one patient with long-standing refractory DCS [35]. Apremilast prescription with the dose of 30 mg twice daily, in this case, resulted in a decrease in the disease flares along with dramatic improvement in clinical manifestations with no adverse events.

### Safety

Biologic and small molecule inhibitors are relatively safe and effective. The adverse events with these agents in DCS patients in our study were as follows: elevated triglycerides and cholesterol levels (1/81), optic neuritis (2/81), infusion reaction (1/81), hepatic cytolysis (1/81), and psoriasiform exanthema (1/81) with TNF-α inhibitor, and eczematous reaction with secukinumab (1/81).

## Discussion

DCS is a chronic, inflammatory, suppurative disease of the scalp, with relapse and remission periods. This disease clinically initiates with folliculitis, with clusters of perifollicular pustules, and then progresses to abscesses and intercommunicating sinus formation, ultimately leading to the development of neutrophilic scarring (cicatricial) alopecia [67, 68]. Apart from the increased risk of bacterial growth, considerable pain, psychological issues, discomfort, and cosmetic problems arising from DCS, squamous cell carcinoma may develop over time from long-standing lesions [67]. Moreover, the refractory nature of the disease, despite various treatments, makes this disease challenging for specialists to manage. Thus, timely diagnosis has a crucial role in effectively managing this therapeutically challenging disease and optimizing patients’ outcomes.

DCS is identified as a part of FOT along with HS, AC, and pilonidal cysts [69]. While DCS is the least prevalent disease of the FOT, it can occur concomitantly with HS and AC, suggesting a similar pathogenic pathway between the diseases [15]. Regardless of the different parts of the body affected by the mentioned diseases, all FOT diseases arise from keratin retention in the follicles of the apocrine gland, leading to pore dilation, bacterial infection, and sinus tract formation [67]. Follicular occlusion can be stimulated by external triggers such as obesity, smoking, and mechanical friction, as well as endogenous factors such as genetic mutations that result in dysregulating keratinocyte differentiation and proliferation [8].

In the absence of evidence-based guidelines, treating DCS is still a therapeutic challenge for dermatologists. Different treatment methods have been utilized alone or in combination to improve the DCS over time, including topical treatment, systemic medication, and invasive modalities [70]. Medical treatments consist of antibiotics, retinoids, steroids, dapsone, and biological therapies [9]. Despite antibiotics and retinoids being the usual treatment protocol for DCS, they are not efficacious enough and have demonstrated a high recurrence rate after treatment cessation [51]. Moreover, invasive modalities, including surgical excision with or without graft, modern external beam radiation therapy, x-ray treatment, and ablative laser therapy of the scalp, were associated with negative sequelae due to their aggressive approach [6, 61]. Concerning previous treatment strategy challenges, immunomodulators are novel treatment agents acting by downregulating the immune system and decreasing inflammatory mediators such as TNF-α, IL-17, and IL-23, which are crucial factors for developing follicular disorders like DCS [9]. Immunomodulatory treatments utilized for managing DCS involve TNF-α blockers, IL inhibitors, JAK inhibitors, and phosphodiesterase inhibitors.

Various TNF-α blockers, including adalimumab, infliximab, golimumab, etanercept, and certolizumab, have been approved for clinical practice in different inflammatory diseases [71]. Adalimumab and infliximab are the most commonly prescribed TNF-α blockers for ameliorating DCS. Adalimumab is the first Food and Drug Administration (FDA)-approved biologic administered as the first-line therapy for moderate-to-severe HS [72]. Off-label treatment of FOT, which carries a similar pathogenesis as DCS, with TNF-α blockers, provided a considerable influence on patients’ outcomes, according to a retrospective study [44]. Also, there have been reported complete responses in two patients and decreased inflammatory symptoms in another patient suffering from DCS after administering TNF-α inhibitors, based on Gamissans et al. investigation [42]. Notably, TNF-α inhibitors were associated with minimized secretion, diminished inflammation, alleviated pain, and improved disease severity scores in most cases suffering from moderate-to-severe DCS who failed on antibiotics and retinoids as well as the patients who developed concomitant HS [5, 32, 39, 43, 45–49, 52–61]. It is imperative to mention that the remaining pathologic residual tissues, such as the interconnecting sinus tract, augment the chance of recurrence in DCS cases receiving conventional options [46]. This was uncommon with TNF-α inhibitor therapy, indicating the lower rates of flare-ups and relapses in treatment with these biologics. Nevertheless, TNF-α inhibitors cannot entirely cure hair loss following DCS, but partial hair regrowth has been documented in some cases [5, 32, 53, 58]. Further, TNF-α inhibitor therapy before surgical excision limited the spread of HS and DCS lesions and provided the basis for faster recovery following surgery [59]. Additionally, adalimumab therapy in a DCS case reduced the serum level of TNF-α and cytokines, such as IL-1RA, IL-1b, and IL-8 [20]. Overall, adalimumab and infliximab were associated with promising outcomes in moderate-to-severe DCS; however, their effectiveness on hair regrowth is still in doubt.

Certolizumab pegol is a novel humanized monoclonal TNF-α inhibitor acting similarly to infliximab and adalimumab [73]. The unique aspect of certolizumab pegol is the absence of fragment crystallizable (Fc) region due to pegylation, which limits antibody-mediated cytotoxicity and passes through the placenta. These findings suggest that certolizumab pegol is a safe choice for pregnant patients suffering from an inflammatory skin condition like HS [74]. In line with previous findings, the application of certolizumab pegol significantly improved the clinical condition of a pregnant patient with DCS without experiencing any adverse effects [62]. However, the data about certolizumab pegol is limited, and further investigations are warranted.

Aside from TNF-α blockers, there have been five reports of the application of IL inhibitors in DCS subjects as the role of IL-17/IL-23 has been established in the pathogenesis of FOT diseases [65, 75]. IL-17 inhibitors, including secukinumab, brodalumab, and ixekizumab, exert a beneficial impact on two-thirds of patients suffering from HS [26, 75, 76]. Administering secukinumab, which contains approval for moderate-to-severe HS, in a patient with DCS ameliorated clinical manifestations [65]. Nonetheless, no data is available regarding other IL-17 inhibitors. Likewise, IL-23 blockers, including guselkumab, tildrakizumab, and risankizumab, all demonstrated favorable outcomes in DCS patients along with improvement of concomitant HS and AC in two patients [63, 64, 66]. These findings were in line with the findings from investigations that evaluated the efficacy of the aforementioned agents in HS patients [77]. Contrariwise, ustekinumab, an IL12/23 blocker, was not correlated with clinical improvement of DCS despite showing desirable results in HS patients [40, 78].

Based on the available evidence, JAK/STATs are critical signaling cascades in a variety of inflammatory diseases [51]. Over 50 soluble factors, such as IL-2, IL-3, IL-4, IL-5, IL-6, and IL-12, as well as interferons, function through a particular composition of JAKs [79]. It has been found that JAK inhibitors selectively disable the ATP-binding site of JAKs, thereby suppressing downstream signaling pathways, which can modulate immune responses under a variety of pathological conditions [80]. Moreover, JAK inhibitors interrupt TNF-α/interferon-γ (IFN-γ) synergy, which induces an inflammatory feedback loop via STAT to minimize hyper-inflammation [81]. Furthermore, a growing body of evidence supports that JAK inhibition influences hair follicle activation and stimulates human dermal papilla cells [82]. This was confirmed by the rapid onset of anagen followed by hair growth in mouse and human skin after administering selective and reversible inhibitors of JAK1 and JAK2 an FDA-approved treatment option for the management of moderate-to-severe alopecia areata, autoimmune non-scarring alopecia [82, 83]. Subsequently, the efficacy and safety findings of JAK inhibitors in HS have depicted a promising prospect of these immunomodulators in treating inflammatory diseases [28, 84, 85]. Recently, utilizing baricitinib in combination with adalimumab in a patient with severe DCS led to obtaining satisfactory outcomes [51]. Further, upadacitinib therapy in another patient with DCS led to dramatic resolution of pustular draining, and bleeding and remarkable improvement in quality of life [34]. Considering the fact that both alopecia areata and DCS are caused by an inflammatory process, it is conceivable that baricitinib may have an impact on DCS’s underlying inflammatory pathway and prevent further scarring [51, 86]. However, it is noteworthy that there is no strong evidence about the impact of JAK inhibitors in reversing the scarring process and inducing hair regrowth, even in other types of CA [86]. Thus, further investigations are recommended to evaluate the efficacy, safety, and mechanism of JAK inhibitors in treating DCS.

Apremilast is another small molecule inhibitor acting through increasing intracellular cyclic adenosine monophosphate by inhibiting PDE4 [87]. Elevated levels of cyclic adenosine monophosphate eventually suppress the secretion of proinflammatory mediators such as TNF-α, IFN-γ, and IL-2 while stimulating the production of the anti-inflammatory cytokine IL-10. Prior evidence illustrated that apremilast can be used as a potential therapeutic option for psoriasis and HS [31, 88]. Similarly, apremilast application in a refractory DCS patient was correlated with notable resolution of disease symptoms and diminution of exacerbations with no adverse events [35].

Regarding safety, our findings indicated that these biologics and small molecule inhibitors were safe with minimized unexpected adverse reactions. The frequency of each adverse event was found to be lower than 2.5% in our study. In a study evaluating adverse reactions following the use of TNF-α inhibitors in VigiAccess of the World Health Organization (WHO), the most reported adverse events of these drugs were infections and infestations (23.0%), musculoskeletal and connective tissue disorders (28.6%), gastrointestinal disorders (15.3%), skin and subcutaneous tissue disorders (13.5%), and nervous system disorders (11.0%) [89]. Besides, the most common adverse reactions of IL-17 inhibitors in psoriasis and psoriatic arthritis patients were as follows: Infection (33.16%), nasopharyngitis (13.74%), and injection site reactions (8.28%) [90]. Our study also showed that the application of small-molecule inhibitors did not result in the occurrence of any adverse events in DCS patients. In line with our findings, the rate of most adverse events did not differ between patients receiving immunomodulators and placebo groups, according to meta-analyses [91–93]. Furthermore, the most prevalent adverse events with IL-17/23 inhibitors and TNF-α blockers in meta-analyses, including injection site reaction, infections, nasopharyngitis, and headache, were not reported in DCS subjects in our study [94, 95]. It is noteworthy that the low frequency or absence of adverse reactions is due to the low prevalence of DCS and limited number of patients in our review.

It is imperative to mention that this study relies on the findings of case reports and cohort studies, which makes it prone to hidden biases. Accordingly, the lack of original studies, the low number of patients due to the disease’s low frequency, and the difficulties of evaluating DCS (relapses versus. remission, inflammation versus. scarring) are limitations that can influence the results. Besides, no study has compared different immunomodulators to gradual treatment protocols for efficacy and safety. Therefore, further research with minimized bias, enhanced power, and a larger scale is needed to verify these results and provide a standardized treatment protocol for DCS. Although many studies have examined different therapeutic options and prepared helpful data for specialists to choose the most effective treatment approach, this study is the only one focusing on immunomodulators due to their potential therapeutic role in candidates suffering from DCS. Furthermore, it is hard to evaluate different components of DCS (relapses vs remission, inflammation vs scarring), which is a common problem in autoinflammatory diseases. Despite the limitations, our study provides valuable information on DCS treatment. Along with all the advantages of immunomodulators, specialists should be aware of the patient’s characteristics and cautious about the increased risk of unwanted adverse events arising from combination therapy to select the most appropriate treatment method for individuals.

## Conclusions

DCS is a chronic, devastating, autoimmune-driven skin disease with no definitive treatment, leading to scar formation. Our systematic review revealed that immunomodulatory drugs are potentially effective for improving DCS lesions in patients suffering from moderate-to-severe DCS, especially in the ones who did not respond to previous treatments. However, achieving a satisfactory treatment response to hair regrowth needs further assessment. Moreover, our study’s reliance on limited data highlights the need for extensive investigations to verify these findings and evaluate the broader landscape of effective treatment options and their adverse events to determine the most effective practice for DCS.

## Supplementary Information

Supplementary material 1

Supplementary material 2

Supplementary material 3

Supplementary material 4

## Data Availability

All relevant data are included in the manuscript and its supplementary files.

## Abbreviations

AC: Acne conglobate
DCS: Dissecting cellulitis of the scalp
DLQI: Dermatology life quality index
Fc: Fragment crystallizable
FDA: Food and Drug Administration
FOT: Follicular occlusion triad or tetrad
HS: Hidradenitis suppurativa
IFN-γ: Interferon-γ
Ig: Immunoglobulin
IL: Interleukin
JAK: Janus kinase
mTOR: Mammalian Target of Rapamycin
PDE: Phosphodiesterase
PGA: Physician’s global assessment
PRISMA: Preferred Reporting Item for Systematic Reviews and Meta-Analysis
STAT: Signal transducers and activators of transcription
Th: T helper
TNF-α: Tumor necrosis factor-α
TYK2: Tyrosine kinase 2
