# Supplementary material for: A systematic review of tumor necrosis factor-α blockers, anti-interleukins, and small molecule inhibitors for dissecting cellulitis of the scalp treatment
Source: Orphanet J Rare Dis. 2025 May 18;20:236. doi: 10.1186/s13023-025-03720-5 (PMC12085841; doi:10.1186/s13023-025-03720-5)
Supplement: Supplementary file 3 — Supplementary material 3 [file 13023_2025_3720_MOESM3_ESM.docx]

**S3 Table**. The list of search strategies and final results on each database.

| **Database**  **(Search date)** | **Step** | **Search strategy** | **Number of results** |
| --- | --- | --- | --- |
| ***PubMed***  ***Feb 4^th^, 2024*** | #1 | **("Perifolliculitis Capitis Abscedens Et Suffodiens, Familial" [Supplementary Concept]) OR ("Dissecting Cellulitis of the Scalp"[Title/Abstract])** | 157 |
|  | #2 | **((((((((((((((((((((((("Interleukin Inhibitors"[Mesh]) OR ("Interleukin Inhibitor*"[Title/Abstract])) OR ("IL Inhibitor*"[Title/Abstract])) OR ("Interleukin 1 Receptor Antagonist Protein"[Mesh])) OR ("anakinra"[Title/Abstract])) OR ("Kineret"[Title/Abstract])) OR ("bermekimab"[Title/Abstract])) OR ("bimekizumab"[Title/Abstract])) OR ("brodalumab"[Title/Abstract])) OR ("canakinumab"[Title/Abstract])) OR ("dupilumab"[Title/Abstract])) OR ("guselkumab"[Title/Abstract])) OR ("ixekizumab"[Title/Abstract])) OR ("risankizumab"[Title/Abstract])) OR ("secukinumab"[Title/Abstract])) OR ("tildrakizumab"[Title/Abstract])) OR ("Ustekinumab"[Title/Abstract])) OR ("Stelara"[Title/Abstract])) OR ("Janus Kinase Inhibitors"[Mesh])) OR ("JAK Inhibitor*"[Title/Abstract])) OR ("Janus Kinase Inhibitor*"[Title/Abstract])) OR ("tofacitinib"[Title/Abstract])) OR ("upadacitinib"[Title/Abstract])) OR ((((((((((((((("Tumor Necrosis Factor Inhibitors"[Mesh]) OR ("Tumor Necrosis Factor Blocker*"[Title/Abstract])) OR ("TNF Inhibitor*"[Title/Abstract])) OR ("TNF Blocker*"[Title/Abstract])) OR ("Tumor Necrosis Factor Antagonist*"[Title/Abstract])) OR ("TNF Antagonist*"[Title/Abstract])) OR ("Adalimumab"[Mesh])) OR ("Adalimumab"[Title/Abstract])) OR ("astegolimab"[Title/Abstract])) OR ("Certolizumab Pegol"[Title/Abstract])) OR ("golimumab"[Title/Abstract])) OR ("Etanercept"[Mesh])) OR ("Etanercept"[Title/Abstract])) OR ("Infliximab"[Mesh])) OR ("Infliximab"[Title/Abstract]))** | 61,041 |
|  | #3 | #1 AND #2 | **22** |
| ***Scopus***  ***Feb 4^th^, 2024*** | #1 | TITLE-ABS-KEY ( "dissecting cellulitis of the scalp" ) OR TITLE-ABS-KEY ( "dissecting cellulitis of scalp" ) OR TITLE-ABS-KEY ( "dissecting folliculitis" ) OR TITLE-ABS-KEY ( "perifolliculitis abscedens et suffodien" ) OR TITLE-ABS-KEY ( "dissecting cellulitis of the scalp" ) | 206 |
|  | #2 | TITLE-ABS-KEY ( "tumor necrosis factor inhibitor*" ) OR TITLE-ABS-KEY ( "anti TNF agent*" ) OR TITLE-ABS-KEY ( "anti tumor necrosis factor agent*" ) OR TITLE-ABS-KEY ( "TNF alpha inhibitor*" ) OR TITLE-ABS-KEY ( "TNF inhibitor*" ) OR TITLE-ABS-KEY ( "Adalimumab" ) OR TITLE-ABS-KEY ( "astegolimab" ) OR TITLE-ABS-KEY ( "Certolizumab Pegol" ) OR TITLE-ABS-KEY ( "golimumab" ) OR TITLE-ABS-KEY ( "Etanercept" ) OR TITLE-ABS-KEY ( "Infliximab" ) OR ( TITLE-ABS-KEY ( "Interleukin Inhibitor*" ) OR TITLE-ABS-KEY ( "IL Inhibitor*" ) OR TITLE-ABS-KEY ( "Interleukin 1 Receptor Antagonist Protein*" ) OR TITLE-ABS-KEY ( "anakinra" ) OR TITLE-ABS-KEY ( "Kineret" ) OR TITLE-ABS-KEY ( "bermekimab" ) OR TITLE-ABS-KEY ( "bimekizumab" ) OR TITLE-ABS-KEY ( "brodalumab" ) OR TITLE-ABS-KEY ( "canakinumab" ) OR TITLE-ABS-KEY ( "dupilumab" ) OR TITLE-ABS-KEY ( "guselkumab" ) OR TITLE-ABS-KEY ( "ixekizumab" ) OR TITLE-ABS-KEY ( "risankizumab" ) OR TITLE-ABS-KEY ( "secukinumab" ) OR TITLE-ABS-KEY ( "tildrakizumab" ) OR TITLE-ABS-KEY ( "Ustekinumab" ) OR TITLE-ABS-KEY ( "Stelara" ) OR TITLE-ABS-KEY ( "Janus Kinase Inhibitor*" ) OR TITLE-ABS-KEY ( "JAK Inhibitor*" ) OR TITLE-ABS-KEY ( "tofacitinib" ) OR TITLE-ABS-KEY ( "upadacitinib" ) ) | 110,791 |
|  | #3 | #1 AND #2 | **37** |
| ***Ovid Embase***  ***Feb 4^th^, 2024*** | #1 | ('dissecting cellulitis of the scalp')/exp OR (("abscessive dissecting head perifolliculitis"):ti,ab,kw) OR (("dissecting cellulitis of scalp"):ti,ab,kw) OR (("dissecting folliculitis"):ti,ab,kw) OR (("perifolliculitis abscedens et suffodien"):ti,ab,kw) OR (("Hoffman dissecting cellulitis"):ti,ab,kw) OR (("Hoffman perifolliculitis"):ti,ab,kw) OR (("dissecting cellulitis of the scalp"):ti,ab,kw) | 253 |
|  | #2 | 'tumor necrosis factor inhibitor'/exp OR 'anti tnf agent*':ti,ab,kw OR 'anti tnf alpha agent*':ti,ab,kw OR 'anti tumor necrosis factor agent*':ti,ab,kw OR 'tnf alpha inhibitor*':ti,ab,kw OR 'tnf inhibitor*':ti,ab,kw OR 'tumor necrosis factor alpha inhibitor*':ti,ab,kw OR 'tumor necrosis factor inhibitor':ti,ab,kw OR 'adalimumab':ti,ab,kw OR 'astegolimab':ti,ab,kw OR 'certolizumab pegol':ti,ab,kw OR 'golimumab':ti,ab,kw OR 'etanercept':ti,ab,kw OR 'infliximab':ti,ab,kw OR 'interleukin inhibitor*':ti,ab,kw OR 'il inhibitor*':ti,ab,kw OR 'interleukin 1 receptor antagonist protein*':ti,ab,kw OR 'anakinra':ti,ab,kw OR 'kineret':ti,ab,kw OR 'bermekimab':ti,ab,kw OR 'bimekizumab':ti,ab,kw OR 'brodalumab':ti,ab,kw OR 'canakinumab':ti,ab,kw OR 'guselkumab':ti,ab,kw OR 'dupilumab':ti,ab,kw OR 'ixekizumab':ti,ab,kw OR 'secukinumab':ti,ab,kw OR 'tildrakizumab':ti,ab,kw OR 'ustekinumab':ti,ab,kw OR 'stelara':ti,ab,kw OR 'janus kinase inhibitor'/exp OR 'janus kinase inhibitor*':ti,ab,kw OR 'jak inhibitor*':ti,ab,kw OR 'janus tyrosine kinase inhibitor*':ti,ab,kw OR 'tofacitinib':ti,ab,kw OR 'upadacitinib':ti,ab,kw | 174,981 |
|  | #3 | #1 AND #2 | **57** |
| **Total inclusion: 116** | | | |
