# Supplementary material for: A systematic review of tumor necrosis factor-α blockers, anti-interleukins, and small molecule inhibitors for dissecting cellulitis of the scalp treatment
Source: Orphanet J Rare Dis. 2025 May 18;20:236. doi: 10.1186/s13023-025-03720-5 (PMC12085841; doi:10.1186/s13023-025-03720-5)
Supplement: Supplementary file 4 — Supplementary material 4 [file 13023_2025_3720_MOESM4_ESM.docx]

| **Study ID (Author, year)** | **NIH Quality Assessment Tool for** **Observational Cohort and Cross-sectional Studies** | | | | | | | | | | | | | | **Quality (Total Quality Score)** |
| --- | --- | --- | --- | --- | --- | --- | --- | --- | --- | --- | --- | --- | --- | --- | --- |
|  | Q1 | Q2 | Q3 | Q4 | Q5 | Q6 | Q7 | Q8 | Q9 | Q10 | Q11 | Q12 | Q13 | Q14 |  |
| **Alzahrani, 2023** | **√** | **√** | **√** | **NR** | **No** | **√** | **√** | **No** | **√** | **√** | **√** | **NR** | **√** | **No** | **9 (Fair)** |
| **Gamissans, 2022** | **√** | **√** | **√** | **NR** | **No** | **√** | **√** | **No** | **√** | **√** | **√** | **NR** | **√** | **No** | **9 (Fair)** |
| **Badaoui, 2016** | **√** | **√** | **√** | **NR** | **No** | **N** | **√** | **No** | **√** | **√** | **√** | **NR** | **√** | **No** | **8 (Fair)** |
| **Sand, 2015** | **√** | **No** | **√** | **No** | **No** | **CD** | **√** | **No** | **No** | **√** | **√** | **NR** | **NR** | **No** | **4 (Poor)** |

**S4 Table:** Quality assessment of observational cohort and cross-sectional studies included in this systematic review.

**Abbreviations:** CD: Cannot determine; NR, not reported

Q1: Was the research question or objective in this paper clearly stated? Q2: Was the study population clearly specified and defined?, Q3: Was the participation rate of eligible persons at least 50%? Q4:  Were all the subjects selected or recruited from the same or similar populations (including the same time period)? Were inclusion and exclusion criteria for being in the study prespecified and applied uniformly to all participants?, Q5: Was a sample size justification, power description, or variance and effect estimates provided?, Q6: For the analyses in this paper, were the exposure(s) of interest measured prior to the outcome(s) being measured?, Q7: Was the timeframe sufficient so that one could reasonably expect to see an association between exposure and outcome if it existed?, Q8:  For exposures that can vary in amount or level, did the study examine different levels of the exposure as related to the outcome (e.g., categories of exposure, or exposure measured as continuous variable)?, Q9: Were the exposure measures (independent variables) clearly defined, valid, reliable, and implemented consistently across all study participants?, Q10: Was the exposure(s) assessed more than once over time?, Q11: Were the outcome measures (dependent variables) clearly defined, valid, reliable, and implemented consistently across all study participants?, Q12: Were the outcome assessors blinded to the exposure status of participants?, Q13: Was loss to follow-up after baseline 20% or less?, Q14: Were key potential confounding variables measured and adjusted statistically for their impact on the relationship between exposure(s) and outcome(s)?

**S5 Table:** Methodological quality assessment in the case series and case reports assessed by Murad et al. checklist.

| **Study ID (Author, year)** | **Selection Bias** | **Ascertainment** | | | **Casualty** | | | **Reporting** | **Total score** |
| --- | --- | --- | --- | --- | --- | --- | --- | --- | --- |
|  | Does the  patient(s)  represent(s)  the whole  experience of  the  investigator  (center) or is  the selection  method  unclear to  the extent  that other  patients with  similar  presentation  may not  have been  reported? | Was the  exposure  adequately  ascertained? | Was the  outcome  adequately  ascertained? | Were other  alternative  causes that  may explain  the  observation  ruled out? | Was there a  challenge/  rechallenge  phenomenon? | Was there a  dose-response  effect? | Was  follow-up  long  enough for  outcomes  to occur? | Is the  case(s)  described  with  sufficient  details to  allow  other investigators  to replicate  the  research or  to allow  practitioners  make  inferences  related to  their own | The total quality score of the included studies |
| **Islam, 2024** | 1 | 1 | 1 | 1 | 0 | 0 | 1 | 1 | 6 |
| **Nagshabandi, 2023** | 1 | 1 | 1 | 1 | 0 | 0 | 1 | 1 | 6 |
| **Bernard, 2023** | 1 | 1 | 1 | 1 | 0 | 0 | 1 | 1 | 6 |
| **Yu, 2023** | 1 | 1 | 1 | 1 | 0 | 0 | 1 | 1 | 6 |
| **Almuhanna, 2023** | 0 | 1 | 1 | 1 | 0 | 0 | 1 | 1 | 5 |
| **Koike, 2022** | 0 | 1 | 1 | 1 | 0 | 0 | 1 | 1 | 6 |
| **Babalola, 2022** | 0 | 0 | 0 | 0 | 0 | 0 | 1 | 1 | 2 |
| **Awad, 2022** | 0 | 0 | 1 | 0 | 0 | 0 | 1 | 1 | 3 |
| **Sanchez-Diaz, 2021** | 1 | 1 | 1 | 1 | 0 | 0 | 1 | 1 | 6 |
| **Spiers, 2021** | 0 | 1 | 1 | 0 | 0 | 0 | 1 | 1 | 4 |
| **Minakawa, 2021** | 1 | 1 | 1 | 1 | 0 | 0 | 1 | 1 | 6 |
| **Kurokawa, 2021** | 0 | 1 | 1 | 0 | 0 | 0 | 1 | 1 | 4 |
| **Frechet, 2021** | 0 | 1 | 1 | 0 | 0 | 0 | 1 | 1 | 4 |
| **De Bedout, 2021** | 0 | 1 | 1 | 0 | 0 | 0 | 1 | 1 | 4 |
| **Alsantali, 2021** | 1 | 1 | 1 | 1 | 0 | 0 | 1 | 1 | 6 |
| **Phillips, 2020** | 0 | 0 | 0 | 0 | 0 | 0 | 1 | 1 | 2 |
| **Muzumdar, 2020** | 0 | 1 | 1 | 0 | 0 | 0 | 1 | 1 | 4 |
| **Maxon, 2020** | 0 | 1 | 1 | 0 | 0 | 0 | 1 | 1 | 4 |
| **Cautela, 2020** | 0 | 1 | 0 | 0 | 0 | 0 | 1 | 1 | 3 |
| **Takahashi, 2019** | 1 | 1 | 1 | 0 | 0 | 0 | 1 | 1 | 5 |
| **Syed, 2018** | 1 | 0 | 0 | 1 | 0 | 0 | 1 | 1 | 4 |
| **Sjerobabski Masnec, 2018** | 1 | 1 | 1 | 1 | 0 | 0 | 1 | 1 | 6 |
| **Mansouri, 2016** | 1 | 1 | 1 | 1 | 0 | 0 | 1 | 1 | 6 |
| **Martin-García, 2015** | 0 | 1 | 1 | 0 | 0 | 0 | 1 | 1 | 4 |
| **Pratsou, 2014** | 0 | 1 | 1 | 0 | 0 | 0 | 1 | 1 | 4 |
| **Lim, 2013** | 1 | 1 | 1 | 1 | 0 | 0 | 1 | 0 | 5 |
| **Wollina, 2012** | 1 | 1 | 1 | 1 | 0 | 0 | 1 | 1 | 6 |
| **Navarini, 2010** | 1 | 1 | 1 | 1 | 0 | 0 | 1 | 1 | 6 |
| **Sukhatme, 2009** | 0 | 1 | 1 | 0 | 0 | 0 | 1 | 1 | 4 |
| **Brandt, 2008** | 1 | 1 | 1 | 1 | 0 | 0 | 1 | 1 | 6 |
